# Supplementary material for: Prospective cohort study evaluating feasibility, acceptability, and clinical impact of diabetes self-management education in a PEN-Plus program in Southeastern Liberia
Source: PLOS Glob Public Health. 2025 Dec 15;5(12):e0005657. doi: 10.1371/journal.pgph.0005657 (PMC12704866; doi:10.1371/journal.pgph.0005657)
Supplement: S1 Data — (ZIP) [file pgph.0005657.s001.zip › DSME A1C data.pdf]

study ID	a1c_0	a1c_3	a1c_6	a1c_12
1	13			
2	9	11	11	17
3	13	8	10	11
4	10	8	10	8
5	13	11	12	11
6	11	11	13	10
7	13	12		
8	13	12		13
9	10		10	11
10	5	6	9	9
11	8	11	9	11
12	14			12
13	14		13	13
14	13	9	9	12
15	13			9
16	13	7		13
17	11	8		9
18	11	9		8
19	13	13	13	14
20	13		12	11
21	13	6		7
22	13	12	12	13
23	13	6	9	14
24	13	9	9	9
25	14		13	8
26	13	6	11	10
