# Supplementary material for: Prospective cohort study evaluating feasibility, acceptability, and clinical impact of diabetes self-management education in a PEN-Plus program in Southeastern Liberia
Source: PLOS Glob Public Health. 2025 Dec 15;5(12):e0005657. doi: 10.1371/journal.pgph.0005657 (PMC12704866; doi:10.1371/journal.pgph.0005657)
Supplement: S1 Data — (ZIP) [file pgph.0005657.s001.zip › DSME Patient knowledge Assessment.pdf]

Pt Study ID	Baseline General_Basic Q1	Baseline General_Basic Q2	Baseline General_Basic Q3	Baseline General_Interm ediateQ1	Baseline BSLevel_Basic Q1
1	0	0	0	0	0
2	0	0	0	0	0
3	2	0	0	2	1
4	0	1	0	1	2
5	0	0	1	1	2
6	0	0	0	0	0
7	0	0	1	1	2
8	2	2	2	2	1
9	1	0	1	2	2
10	0	0	0	1	1
11	0	0	0	0	1
12	0	0	1	1	1
13	0	0	0	1	1
14	0	0	1	1	1
15	0	1	1	1	1
16	0	0	1	0	1
17	1	0	2	2	2
18	0	0	0	1	1
19	0	0	1	1	1
20	0	0	0	0	0
21	0	0	1	1	2
22	0	0	1	1	1
23	0	0	1	2	0
24	0	0	1	1	1
25	0	0	1	1	2
26	0	0	0	1	1

Baseline BSLevel_BasicQ2	Baseline BSLevel_BasicQ3	Baseline BSLevel_BasicQ4	Baseline BSLevel_IntermediateQ1	Baseline BSLevel_IntermediateQ2	Baseline BSLevel_IntermediateQ3
0	0	0	0	0	0
0	0	0	0	0	0
2	2	2	2	2	2
2	1	2	2	1	2
0	1	1	1	2	0
0	0	0	0	0	0
0	1	1	1	2	0
2	1	2	2	2	2
1	2	2	1	2	2
0	2	1	1	2	1
1	0	2	1	1	2
1	1	1	0	1	0
1	1	1	0	0	0
1	1	1	1	1	1
1	1	1	1	0	1
1	1	1	1	0	1
1	2	1	1	2	2
1	1	1	1	1	1
1	1	1	1	0	1
0	0	0	0	0	0
0	1	0	2	0	2
1	1	1	1	0	0
0	0	0	0	0	0
1	1	1	1	0	1
2	2	2	2	0	1
1	1	1	0	1	0

Baseline	Baseline	Baseline	Baseline	Baseline	Baseline
BSLevel_IntermediateQ4	BSMonitoring_BasicQ1	BSMonitoring_BasicQ2	BSMonitoring_BasicQ3	BSMonitoring_BasicQ4	BSMonitoring_BasicQ5
0	0	0	0	0	0
0	0	0	0	0	0
2	2	2	0	1	1
2	2	2	2	2	2
1	1	2	2	0	2
0	0	0	0	0	0
1	1	2	2	0	2
2	2	2	2	1	2
2	2	2	2	2	2
0	0	1	1	1	1
1	0	0	1	1	1
1	2	0	1	1	1
0	0	0	0	0	0
1	0	1	0	0	1
1	0	1	2	1	1
1	0	0	0	0	0
2	0	1	2	2	2
1	0	0	0	0	1
1	0	0	0	0	1
0	0	0	0	0	0
0	0	0	0	0	0
1	0	0	0	0	1
0	0	0	0	0	0
1	0	0	0	0	1
1	2	0	0	0	0
1	2	0	1	1	1

Baseline Insulin_BasicQ1	Baseline Insulin_BasicQ2	Baseline Insulin_BasicQ3	Baseline Insulin_BasicQ4	Baseline Insulin_BasicQ5	Baseline Insulin_BasicQ6
0	0	0	0	0	0
0	0	0	0	0	0
2	1	1	2	1	1
2	2	2	0	2	2
1	2	1	1	2	2
0	0	0	0	0	0
1	2	1	1	2	2
2	2	1	2	2	2
2	2	0	2	2	2
0	1	0	2	2	1
1	0	1	0	2	2
2	1	0	2	2	2
2	1	0	2	1	1
2	1	0	2	1	1
2	1	0	2	1	1
2	1	0	2	1	1
2	1	1	2	2	2
2	1	0	0	0	1
2	1	0	2	1	1
1	1	0	2	1	1
2	0	0	0	0	0
2	1	0	2	1	1
0	0	0	0	0	
2	1	0	1	1	1
2	2	2	2	2	2
2	1	0	2	2	2

Baseline Insulin_BasicQ7	Baseline Insulin_IntermediateQ1	Baseline Insulin_IntermediateQ2	Baseline Insulin_IntermediateQ3	Baseline Diet_BasicQ1	Baseline Diet_BasicQ2
0	0	0	0	0	0
0	0	0	0	0	0
2	0	0	2	2	2
2	2	2	2	2	2
2	1	2	0	1	2
0	0	0	0	0	0
2	1	2	0	1	2
2	1	2	0	2	1
2	2	2	2	2	2
1	0	0	0	2	1
1	1	0	0	0	2
1	1	1	0	1	1
2	2	0	0	2	0
2	0	0	0	1	1
2	0	0	0	1	1
2	0	0	0	2	1
2	2	0	1	2	1
1	0	0	0	1	1
2	0	0	0	1	0
0	0	0	0	1	1
0	0	0	0	0	0
2	0	0	0	1	1
0	0	0	0	0	0
1	0	0	0	1	1
2	2	2	2	2	2
1	1	1	1	1	0

Baseline Diet_IntermediateQ1	Baseline Diet_IntermediateQ2	Baseline Complications_BasicQ1	Baseline Complications_BasicQ2	Baseline Complication_BasicQ3	Baseline Complications_IntermediateQ1
0	0	0	0	0	0
0	0	0	0	0	0
0	0	0	1	1	1
2	1	0	2	2	2
0	0	2	0	1	1
0	0	0	0	0	0
0	0	2	0	1	1
0	0	1	1	2	2
1	0	2	2	2	1
0	0	0	1	1	0
1	0	0	0	1	1
0	1	0	0	0	0
0	0	0	0	1	0
0	0	1	1	1	1
0	0	1	1	2	1
0	0	1	0	1	0
1	1	2	1	0	0
0	0	1	1	1	0
0	0	1	1	1	1
0	0	1	0	1	0
0	0	0	0	0	0
0	0	0	0	1	1
0	0	0	0	0	0
0	0	1	1	1	1
2	0	0	2	2	0
0	0	1	1	0	0

Baseline Complications_IntermediateQ2	Baseline Sick_BasicQ1	Baseline Sick_BasicQ2	Baseline Sick_BasicQ3	Baseline Sick_BasicQ4	Baseline Sick_IntermediateQ1
0	0	0	0	0	0
0	0	0	0	0	0
1	0	1	1	1	1
1	0	0	2	2	2
0	0	0	1	1	1
0	0	0	0	0	0
0	0	0	1	1	1
1	1	2	1	1	1
0	0	2	2	2	2
0	0	0	0	1	1
0	0	0	0	0	1
0	0	0	0	0	0
0	0	0	0	0	0
1	0	1	1	1	0
0	0	1	1	1	0
0	0	1	1	1	0
1	0	1	1	1	0
0	0	1	1	1	0
0	0	1	1	0	0
0	0	1	1	0	0
0	0	0	0	0	0
0	0	1	1	0	0
0		0	0	0	0
2	0	1	1	0	0
0	0	2	1	0	2
0	0	0	0	0	0

Baseline Sick_IntermediateQ2	Baseline Activity_BasicQ1	Baseline Activity_BasicQ2	Baseline Activity_BasicQ3	Baseline Activity_BasicQ4	Baseline Activity_IntermediateQ1
0	0	0	0	0	0
0	0	0	0	0	0
0	1	0	0	0	0
2	1	2	2	2	0
0	0	1	0	0	1
0	0	0	0	0	0
0	0	1	0	0	1
2	2	1	1	1	1
1	2	2	2	0	2
1	1	0	1	1	1
1	1	1	0	1	1
0	0	0	0	0	0
0	0	0	0		0
1	1	0	0	1	0
1	1	0	0	0	0
1	1	0	0	0	0
0	2	1	0	0	1
0	1	0	0	0	0
0	1	1	0	1	0
0	1	0	0	0	0
0	0	0	0	0	0
0	1	0	0	0	0
0	0	0	0	0	0
1	1	0	0	0	0
0	2	2	2	0	0
0	1	0	0	0	0

Baseline	Baseline	Baseline	Baseline	Baseline	Baseline
Activity_IntermediateQ2	Maintenance_BasicQ1	Maintenance_BasicQ2	Maintenance_IntermediateQ1	FemalesQ1	FemalesQ2
0	0	0	0	0	0
0	0	0	0	0	0
0	0	0	0	0	0
2	0	0	0	1	na
1	1	0	0	na	na
0	0	0	0	0	0
1	1	0	0	na	na
1	2	0	2	0	0
0	0	0	1	na	na
0	0	na	0	0	0
1	0	0	na	0	0
0	0	0	0	0	0
0	0	0	1	na	na
0	0	0	1	0	0
0	0	0	1	0	0
1	0	0	0	0	0
0	0	0	1	na	na
0	0	0	1	0	0
0	0	0	0	0	0
0	0	0	0	0	0
0	0	0	0	0	0
0	0	0	0	na	na
0	0	0	0	0	0
0	0	0	1	0	0
2	0	0	0	0	0
0	0	0	0	na	na

6 month<br>General_Basic Q1	6 month<br>General_Basic Q2	6 month<br>General_Basic Q3	6 month<br>General_IntermediateQ1	6 month<br>BSLevel_Basic Q1	6 month<br>BSLevel_Basic Q2
0	0	0	0	0	0
2	2	2	2	2	2
0	0	2	2	1	2
1	1	2	2	2	2
2	2	2	2	2	2
0	0	2	1	2	2
2	2	2	2	2	2
2	1	2	2	2	0
0	1	2	2	2	2
0	0	2	2	2	2
2	0	2	0	2	2
0	0	1	0	1	1
0	0	1	2	2	2
0	0	1	1	0	2
2	1	1	0	2	1
0	1	2	1	1	2
0	0	0	0	2	2
0	0	1	0	2	1
0	0	2	1	1	2
0	0	0	2	1	1
0	0	1	1	1	1
0	0	1	0	2	1
1	1	2	2	2	2
1	1	2	2	2	2
2	2	2	2	2	2

6 month BSLevel_BasicQ3	6 month BSLevel_BasicQ4	6 month BSLevel_IntermediateQ1	6 month BSLevel_IntermediateQ2	6 month BSLevel_IntermediateQ3	6 month BSLevel_IntermediateQ4
0	0	0	0	0	0
2	2	2	2	2	2
2	2	2	2	2	2
2	2	2	1	2	2
2	2	2	2	2	2
2	2	2	0	2	2
2	2	2	2	2	2
2	2	2	2	2	2
2	0	2	0	2	2
2	2	2	0	2	2
2	2	2	2	2	2
1	0	0	0	1	0
2	2	2	2	2	2
2	1	2	1	1	1
2	1	1	0	1	1
1	1	1	1	1	2
2	1	1	1	1	1
2	2	1	0	2	2
2	2	1	0	0	1
2	1	1	1	1	0
1	1	1	0	0	1
1	1	1	0	2	1
2	2	2	2	2	2
2	2	2	2	2	2
2	2	2	2	0	0

6 month BSMonitoring_BasicQ1	6 month BSMonitoring_BasicQ2	6 month BSMonitoring_BasicQ3	6 month BSMonitoring_BasicQ4	6 month BSMonitoring_BasicQ5	6 month Insulin_BasicQ1
0	0	0	0	0	0
2	2	2	2	2	2
2	2	2	2	2	2
2	1	2	2	2	2
2	2	2	2	2	2
2	2	2	2	2	2
2	2	2	2	2	2
2	2	2	2	2	2
2	2	2	2	2	2
2	2	0	0	0	0
2	2	1	0	0	2
0	0	0	0	0	2
2	2	2	2	2	2
1	1	1	2	1	2
2	1	0	2	1	2
2	1	2	2	2	2
0	1	2	2	0	2
0	1	1	1	0	2
0	1	0	0	0	2
0	0	0	0	0	0
0	0	0	0	1	2
2	0	0	1	2	2
1	2	1	2	2	2
2	2	2	1	2	2
0	0	1	1	1	1

6 month Insulin_BasicQ2	6 month Insulin_BasicQ3	6 month Insulin_BasicQ4	6 month Insulin_BasicQ5	6 month Insulin_BasicQ6	6 month Insulin_BasicQ7
0	0	0	0	0	0
2	2	2	2	2	2
2	2	2	2	2	2
2	2	2	2	2	2
2	2	2	2	2	2
2	1	2	2	2	2
2	2	2	2	2	2
2	2	2	2	2	2
2	2	2	2	2	2
0	0	0	0	0	0
2	2	2	2	2	2
1	1	2	1	2	2
2	2	2	2	2	2
1	1	2	1	2	1
2	1	2	1	1	1
2	2	2	2	2	2
1	1	2	1	1	1
1	1	2	1	1	1
2	1	2	1	2	2
0	0	0	0	1	1
1	0	2	1	1	2
1	1	2	1	2	1
1	1	2	2	2	2
1	1	2	2	2	2
1	1	1	2	2	2

6 month<br><br>Insulin_IntermediateQ1	6 month<br><br>Insulin_IntermediateQ2	6 month<br><br>Insulin_IntermediateQ3	6 month<br><br>Diet_BasicQ1	6 month<br><br>Diet_BasicQ2	6 month<br><br>Diet_IntermediateQ1
0	0	0	0	0	0
2	2	2	2	2	2
2	2	2	2	2	0
2	1	1	2	2	0
2	1	2	2	2	2
2	2	2	2	2	0
2	2	2	2	2	2
2	2	2	2	2	2
2	2	2	2	2	2
0	0	0	2	0	0
2	1	2	2	2	0
1	1	1	1	1	1
2	2	2	2	2	2
1	0	1	1	0	0
2	0	0	0	0	2
2	0	2	2	2	2
1	0	1	1	1	0
1	1	1	1	1	0
1	1	2	1	1	0
0	0	0	0	0	0
0	0	0	1	1	0
1	2	1	1	1	0
2	1	2	2	1	2
2	1	2	2	2	1
2	2	2	2	2	1

6 month Diet_IntermediateQ2	6 month Complications_BasicQ1	6 month Complications_BasicQ2	6 month Complication_BasicQ3	6 month Complications_IntermediateQ1	6 month Complications_IntermediateQ2
0	0	0	0	0	0
2	2	2	2	2	2
0	0	0	0	2	1
0	1	2	2	2	1
2	2	2	2	2	2
0	2	0	0	0	0
2	2	2	2	2	2
1	2	2	2	1	2
2	2	2	2	2	2
0	0	2	1	0	0
1	1	2	2	1	2
0	0	0	0	0	0
2	2	1	2	1	1
0	1	1	1	0	0
1	0	0	2	0	0
1	1	1	1	0	0
0	0	1	1	1	0
0	0	1	1	1	0
0	0	1	1	1	0
0	0	0	0	0	0
0	0	0	1	1	0
0	0	1	2	2	1
2	2	2	1	2	2
1	2	2	2	2	2
2	2	2	2	1	2

6 month<br>Sick_BasicQ1	6 month<br>Sick_BasicQ2	6 month<br>Sick_BasicQ3	6 month<br>Sick_BasicQ4	6 month<br>Sick_IntermediateQ1	6 month<br>Sick_IntermediateQ2
0	0	0	0	0	0
2	2	2	2	2	2
0	0	0	0	2	2
1	2	2	2	1	1
2	2	2	2	2	2
0	0	0	0	0	0
2	2	2	2	2	2
0	0	2	1	1	2
0	0	0	0	2	2
0	0	0	0	0	0
2	0	1	1	1	1
0	0	0	0	0	0
1	1	1	1	1	1
1	1	1	1	1	1
0	2	1	1	2	1
0	2	2	1	2	2
1	1	1	1	1	1
0	0	1	1	1	0
0	0	0	0	0	0
0	0	0	0	0	0
0	1	1	0	0	0
0	0	1	1	2	1
0	2	2	2	2	1
0	2	2	2	2	1
2	2	0	0	0	0

6 month Activity_BasicQ1	6 month Activity_BasicQ2	6 month Activity_BasicQ3	6 month Activity_BasicQ4	6 month Activity_IntermediateQ1	6 month Activity_IntermediateQ2
0	0	0	0	0	0
2	2	2	2	2	2
2	2	2	2	2	2
2	2	2	1	1	2
2	2	2	2	2	2
1	1	1	1	0	1
2	2	2	2	2	2
2	2	2	1	2	2
2	2	1	1	1	1
0	0	0	0	0	0
	1	1	1	1	1
0	0	0	0	0	0
2	2	2	2	2	1
2	1	1	1	1	1
2	1	1	1	0	0
2	2	1	0	0	2
1	1	1	1	0	0
1	0	0	0	0	0
0	0	0	0	0	0
0	0	0	0	0	0
1	0	0	0	0	0
2	1	1	0	0	0
2	1	1	2	1	2
2	1	1	2	1	2
0	0	0	0	0	0

6 month<br>Maintenance_BasicQ1	6 month<br>Maintenance_BasicQ2	6 month<br>Maintenance_IntermediateQ1	6 month<br>FemalesQ1	6 month<br>FemalesQ2	12 month<br>General_BasicQ1
0	0	0	0	0	0
	2	2	0	0	
0	0	0	na	na	0
0	1	2	na	na	2
2	2	0	na	na	1
1	0	0	na	na	
2	2	2	0	0	2
2	2	1	na	na	
1	1	1	0	0	0
0		0	0	0	0
1	1	1	0	0	
0	0	0	0	0	0
1	1	1	0	0	0
1	1	1	1	1	2
0	0	1	na	na	2
2	2	1	0	0	1
0	1	0	0	0	1
0	1	1	0	0	1
0	0	0	0	0	0
0	0	0	0	0	0
0	0	0	na	na	0
0	0	1	0	0	0
0	0	1	0	0	2
0	0	1	0	0	1
0	0	0	na	na	0

12 month<br>General_Basic Q2	12 month<br>General_Basic Q3	12 month<br>General_IntermediateQ1	12 month<br>BSLevel_Basic Q1	12 month<br>BSLevel_Basic Q2	12 month<br>BSLevel_Basic Q3
0	2	2	2	2	2
0	1	2	2	2	2
1	2	2	2	2	2
1	2	2	2	2	2
2	2	2	2	2	2
0	1	0	2	2	2
0	2	2	2	2	2
0	1	0	1	2	2
0	1	2	2	2	2
2	2	1	2	2	2
2	2	1	2	2	2
1	2	2	2	2	2
1	1	1	1	1	1
0	1	1	2	2	2
0	1	1	1	1	1
0	1	0	1	1	1
0	1	0	1	1	1
0	2	0	1	1	1
0	1	1	1	1	1
2	2	2	2	1	2
0	0	0	0	0	1

12 month BSLevel_Basic Q4	12 month BSLevel_IntermediateQ1	12 month BSLevel_IntermediateQ2	12 month BSLevel_IntermediateQ3	12 month BSLevel_IntermediateQ4	12 month BSMonitoring_BasicQ1
2	2	1	2	2	1
1	1	1	0	2	2
2	2	2	2	2	2
2	2	2	2	2	2
2	2	2	2	2	2
1	1	2	1	2	2
2	2	1	2	2	1
1	1	1	1	1	1
2	1	1	1	2	2
2	2	2	2	2	1
2	2	2	2	2	2
2	2	2	2	2	2
1	1	1	1	1	2
1	2	1	2	2	2
1	2	1	1	2	1
1	1	1	1	1	1
1	1	0	1	1	1
1	1	1	1	1	1
1	1	1	1	1	1
2	2	2	2	2	2
1	0	0	0	0	0

12 month BSMonitoring_BasicQ2	12 month BSMonitoring_BasicQ3	12 month BSMonitoring_BasicQ4	12 month BSMonitoring_BasicQ5	12 month Insulin_BasicQ1	12 month Insulin_BasicQ2
0	2	2	2	2	0
0	0	1	1	2	1
2	2	2	2	2	2
2	2	1	1	2	2
2	2	2	2	2	2
1	1	2	2	2	2
0	2	2	1	1	0
1	1	1	2	2	2
2	2	2	2	2	2
2	2	2	2	2	2
2	2	2	2	2	2
2	2	1	1	1	2
1	1	1	1	1	1
1	1	1	1	2	2
1	1	1	1	2	0
1	1	1	1	12	0
2	2	0	0	2	1
0	1	0	1	1	1
1	2	1	1	1	1
1	2	2	2	2	2
0	0	0	0	1	1

12 month Insulin_BasicQ3	12 month Insulin_BasicQ4	12 month Insulin_BasicQ5	12 month Insulin_BasicQ6	12 month Insulin_BasicQ7	12 month Insulin_IntermediateQ1
1	2	2	2	2	2
1	2	1	1	2	1
2	2	2	2	2	2
0	2	2	2	2	2
1	2	2	2	2	2
2	2	2	2	2	2
0	2	0	2	0	2
2	2	2	2	2	2
0	2	2	2	2	2
2	2	2	2	2	2
2	1	2	2	2	2
2	2	1	2	1	2
1	1	0	0	0	0
1	2	2	2	2	2
1	2	2	2	2	1
1	2	0	2	1	1
0	1	1	1	1	1
1	2	1	2	1	2
1	1	1	1	1	1
1	2	2	2	2	2
1	1	1	1	1	1

12 month Insulin_IntermediateQ2	12 month Insulin_IntermediateQ3	12 month Diet_BasicQ1	12 month Diet_BasicQ2	12 month Diet_IntermediateQ1	12 month Diet_IntermediateQ2
2	2	2	2	2	2
0	0	2	1	1	2
2	2	2	2	2	2
2	2	2	2	1	0
2	2	2	2	2	2
2	2	2	2	2	2
0	0	2	0	0	0
2	1	1	1	1	1
2	2	0	0	0	1
2	2	2	2	2	2
2	1	1	1	1	2
2	1	1	1	1	1
0	0	1	1	1	1
2	2	1	2	0	1
2	1	1	0	1	0
0	0	1	1	0	0
2	2	1	1	1	1
1	1	1	1	2	0
1	1	1	1	2	2
2	2	2	2	2	2
1	0	0	1	0	0

12 month Complications_BasicQ1	12 month Complications_BasicQ2	12 month Complication_BasicQ3	12 month Complications_IntermediateQ1	12 month Complications_IntermediateQ2	12 month Sick_BasicQ1
2	2	2	2	1	1
2	2	1	0	0	0
2	2	2	2	2	1
2	2	1	1	2	0
2	2	2	2	2	2
2	2	2	2	2	0
2	2	1	0	0	0
1	1	1	1	1	0
2	2	2	2	2	2
1	0	2	1	1	2
2	2	2	2	2	2
1	1	2	2	1	1
1	0	0	0	0	0
0	0	0	0	0	0
0	1	1	0	0	0
0	0	0	0	1	1
1	1	1	0	0	0
0	0	0	0	2	0
2	2	1	1	1	1
2	2	2	2	2	2
0	0	0	0	0	0

12 month	12 month	12 month	12 month	12 month	12 month
Sick_BasicQ2	Sick_BasicQ3	Sick_BasicQ4	Sick_IntermediateQ1	Sick_IntermediateQ2	Activity_BasicQ1
1	1	2	2	2	12
1	2	2	1	2	1
2	2	2	1	2	2
2	2	2	2	2	2
2	2	2	2	2	2
2	2	2	2	2	2
2	1	1	0	1	2
1	1	1	1	1	1
2	1	2	2	2	2
1	2	2	2	2	2
2	2	2	2	2	2
1	1	1	1	1	1
0	0	0	0	0	0
0	0	0	0	0	0
1	1	1	1	1	1
1	0	0	0	0	0
1	1	2	2	2	1
0	0	1	2	2	1
1	2	2	2	2	2
2	2	2	2	2	2
0	0	0	0	0	0

12 month	12 month	12 month	12 month	12 month	12 month
Activity_BasicQ2	Activity_BasicQ3	Activity_BasicQ4	Activity_IntermediateQ1	Activity_IntermediateQ2	Maintenance_BasicQ1
2	1	1	1	1	1
1	1	2	2	0	0
2	2	1	2	2	1
2	2	1	2	2	0
2	2	2	2	2	2
2	2	1	1	2	0
2	2	0	1	1	0
0	1	1	1	1	0
2	2	2	2	2	2
1	2	2	2	2	0
2	2	2	1	1	1
1	1	1	1	1	1
0	1	1	1	1	1
0	0	0	0	0	0
1	1	1	1	1	1
0	0	0	1	1	1
1	1	1	0	2	0
2	2	0	0	0	0
2	2	2	1	1	1
2	2	2	2	2	2
0	0	0	0	0	0

12 month Maintenance_BasicQ2	12 month Maintenance_IntermediateQ1	12 month FemalesQ1	12 month FemalesQ2
1	1	1	2
0	0	0	0
0	1	na	na
0	2	na	na
2	2	2	2
0	2	2	1
0	0	0	0
0	1	0	0
2	2	2	1
1	1	1	1
1	1	0	0
1	1	na	na
1	1	0	0
0	0	0	0
1	0	0	1
1	1	0	0
0	2	2	1
0	1	2	2
1	1	0	0
0	0	0	0
0	0	na	na
