# Supplementary material for: Prospective cohort study evaluating feasibility, acceptability, and clinical impact of diabetes self-management education in a PEN-Plus program in Southeastern Liberia
Source: PLOS Glob Public Health. 2025 Dec 15;5(12):e0005657. doi: 10.1371/journal.pgph.0005657 (PMC12704866; doi:10.1371/journal.pgph.0005657)
Supplement: S1 Data — (ZIP) [file pgph.0005657.s001.zip › DSME-MonthlyDocumentation.pdf]

Visit Numb	Patient SM	# times in t	# times in p	Brought glu	Brought log	In the past week, # times BG recor
1						
1	1	N/A or blar	5	5 Yes	Yes	7
1	2	Yes	6	6 No	No	0
1	3	No	7	7 Yes	No	0
1	4	No	7	7 Yes	Yes	0
1	5	Yes	7	7 Yes	Yes	14
1	6	No	7	7 Yes	Yes	0
2						
2	1	Yes	0	0 Blank	Blank	0
2	2	Yes	6	6 No	No	0
2	3	Yes	5	4 Yes	No	0
2	4	Yes	4	3 Yes	No	0
2	5	Yes	7	7 Yes	No	0
2	6	No	7	7 No	No	0
2	8	No	7	7 No	No	0
2	9	No	0	0 No	No	0
2	10	Yes	7	7 No	No	0
2	12	Yes	7	7 No	No	0
3						
3	1	Yes	4	3 Yes	Yes	10
3	2	Yes	7	7 Yes	Yes	28
3	3	Yes	7	7 Yes	Yes	13
3	4	Yes	7	7 Yes	Yes	14
3	5	Yes	7	7 Yes	No	0
3	6	Yes	7	7 No	Yes	14
3	7	Yes	7	7 Yes	Yes	14
4						
4	1	Yes	6	5 Yes	Yes	14
4	2	Yes	7	0 Yes	Yes	9
4	3	Yes	5	0 Yes	Yes	9
4	4	No	4	0 Yes	No	10
4	5	Yes	0	0 Yes	Yes	14
4	6	Yes	7	7 Yes	Yes	14
4	7	Yes	7	7 Yes	Yes	14
4	8	No	0	0 No	No	0
4	9	Yes	7	7 Yes	Yes	14
4	10	Yes	7	7 Yes	Yes	14
4	11	Yes	7	7 Yes	Yes	14
4	12	N/A or blar	7	7 Yes	Yes	14
5						
5	1	Yes	6	0 Yes	Yes	4
5	2	N/A or blar	7	3 Yes	No	0
5	3	Yes	7	3 Yes	Yes	0

5	4	Yes	7	7	Yes	Yes	9
5	6	Yes	7	7	Yes	Yes	14
5	7	Yes	7	7	Yes	Yes	14
5	8	No	7	7	Yes	Yes	14
5	10	Yes	7	7	Yes	No	0
5	11	Yes	7	7	Yes	Yes	14
5	12	No	7	7	Yes	Yes	14
6							
6	1	N/A or blar	6	6	Yes	Yes	10
6	2	Yes	7	7	No	No	0
6	3	Yes	7	7	Yes	Yes	14
6	4	Yes	7	7	Yes	Yes	13
6	7	Yes	7	7	Yes	Yes	14
6	6	Yes	7	7	Yes	Yes	14
6	5	Yes	7	7	Yes	Yes	14
6	8	Yes	7	7	Yes	Yes	14
6	9	No	7	7	Yes	Yes	14
6	10	Yes	7	7	Yes	Yes	14
6	11	Yes	7	7	Yes	Yes	14
6	12	Yes	7	7	Yes	Yes	14
7							
7	1	N/A or blar	7	7	No	Yes	0
7	2	Yes	7	7	No	No	0
7	3	Yes	7	7	No	No	
7	4	N/A or blar	7	7	No	No	0
7	5	Yes	7	7	Yes	No	0
7	6	Yes	7	7	No	Yes	14
7	7	No	7	7	Yes	Yes	14
7	8	No	0	0	Yes	Yes	14
7	9	No	7	7	Yes	Yes	14
7	10	Yes	7	7	No	No	0
7	11	Yes	7	7	No	No	0
7	12	Yes	7	7	No	No	0
8							
8	1	Yes			Blank	Yes	2
8	2	Yes	7	6	No	Yes	14
8	3	Yes	7	7	Yes	Yes	14
8	4	Yes	7	7	Yes	Yes	12
8	5	Yes	7	7	Yes	Yes	14
8	6	Yes	7	7	Yes	Yes	14
8	7	Yes	7	7	Yes	Yes	14
8	8	Yes	7	7	Yes	Yes	14
8	9	No	7	7	Yes	Yes	14
8	10	Yes	7	7	Yes	Yes	14

8	11	Yes	7	7	Yes	Yes	14
8	12	Yes	7	7	Yes	Yes	14
9							
9	1	Yes	7	7	No	No	0
9	4	Yes	5	4	Yes	No	0
9	7	Yes	0	0	Yes	Yes	0
9	8	N/A or blar	5	6	Yes	Yes	0
9	9	Yes	7	7	Yes	No	0
9	10	Yes	7	7	No	Yes	10
9	11	Yes	4	7	Yes	Yes	0
10							
10	1	No	5	7	No	Yes	0
10	2	Yes	6	6	Yes	Yes	14
10	3	Yes	7	7	Yes	No	0
10	4	Yes	7	7	Yes	Yes	13
10	5	Yes	7	7	Yes	Yes	14
10	6	Yes	7	7	Yes	Yes	14
10	7	Yes	7	7	Yes	No	0
10	8	No	0	0	No	No	0
10	9	No	7	7	Yes	Yes	0
10	10	Yes	7	7	Yes	Yes	14
10	11	No	7	7	Yes	No	0
10	12	Yes	7	7	Yes	Yes	14
11							
11	1	Yes	0	2	No	No	0
11	2	N/A or blar	6	6	Yes	Yes	12
11	3	Yes	7	7	Yes	No	0
11	6	Yes	7	7	Yes	Yes	14
11	4	Yes	7	7	Yes	No	0
11	7	No	7	7	Yes	No	0
11	9	No	7	7	Yes	Yes	14
11	10	Yes	7	7	Yes	Yes	14
11	11	No	7	7	Yes	Yes	14
11	12	Yes	7	7	Yes	Yes	14
12							
12	1	No	4	4	Yes	No	4
12	2	Yes	7	7	Yes	Yes	14
12	3	N/A or blar	7	7	Yes	Yes	14
12	4	Yes	7	7	No	Yes	14
12	5	Yes	6	6	Yes	Yes	13
12	7	Yes	7	7	Yes	Yes	14
12	8	Yes	7	7	Yes	Yes	14
12	9	Yes	7	2	Yes	Yes	14
12	10	Yes	7	7	Yes	Yes	14

12	11	Yes	7	7 Yes	Yes	14
12	12	No	7	7 No	No	0
13						
13	1	No	5	5 No	No	0
13	2	No	2	2 Yes	No	0
13	3	No	5	5 Yes	Yes	0
13	4	Yes	4	4 Yes	No	0
13	5	No	5	5 Yes	Yes	7
13	6	No	4	4 Yes	No	6
13	7	Yes	4	4 Yes	Yes	0
13	8	No	4	2 Yes	No	0
13	9	Yes	4	4 Yes	Yes	7
13	10	Yes	6	6 Yes	No	13
13	11	Yes	4	4 Yes	Yes	7
13	12	Yes	7	7 Yes	Yes	14
14						
14	1	No	7	7 Yes	No	0
14	2	Yes	7	7 Yes	No	0
14	3	Yes	7	7 Yes	No	0
14	4	Yes	7	6 Yes	Yes	12
14	5	Yes	7	7 Yes	Yes	14
14	6	No	6	6 Yes	No	12
14	7	Yes	6	4 Yes	No	12
14	8	Yes	7	7 Yes	Yes	14
14	9	Yes	7	7 Yes	Yes	14
14	10	Yes	7	7 Yes	Yes	14
14	11	Yes	7	7 Yes	Yes	14
14	12	No	7	7 No	No	0
15						
15	1	N/A or blar	7	7 Yes	Yes	10
15	2	Yes	4	4 Yes	Yes	9
15	3	Yes	4	4 Yes	No	0
15	4	Yes	4	2 Yes	No	0
15	6	No	6	6 Yes	Yes	14
15	7	No	6	6 Yes	Yes	11
15	8	No	6	4 Yes	Yes	12
15	9	No	7	7 Yes	Yes	14
15	10	Yes	7	7 Yes	Yes	14
15	11	Yes	4	2 Yes	Yes	0
15	12	Yes	5	5 No	No	0
16						
16	7	No	4	4 Yes	Yes	14
16	1	Yes	7	6 Yes	Yes	13
16	2	Yes	7	7 Yes	Yes	14

16	3	Yes	6	6 Yes	Yes	0
16	4	Yes	4	4 Yes	Yes	10
16	5	Yes	4	4 Yes	Yes	14
16	6	No	5	4 Yes	No	0
16	8	No	6	6 Yes	Yes	0
16	9	Yes	7	7 Yes	No	14
16	10	Yes	4	4 Yes	Yes	14
16	11	Yes	5	5 Yes	Yes	14
17						
17	1	Yes	7	7 Yes	No	0
17	4	Yes	4	4 No	No	0
17	6	No	6	6 Yes	No	0
17	8	No	6	6 Yes	No	0
17	9	Yes	4	7 Yes	Yes	14
17	10	Yes	7	7 Yes	Yes	14
17	11	No	2	2 Yes	Yes	14
18						
18	1	Yes	6	6 Yes	No	0
18	2	Yes	7	7 Yes	Yes	14
18	3	Yes	5	4 Yes	Yes	13
18	4	Yes	6	3 Yes	Yes	10
18	5	Yes	5	5 Yes	Yes	6
18	6	No	5	4 Yes	No	10
18	7	Yes	6	4 Yes	No	7
18	8	Yes	7	7 Yes	No	14
18	9	Yes	7	7 Yes	Yes	14
18	10	Yes	7	7 Yes	Yes	13
18	11	Yes	5	5 Yes	Yes	14
18	12	Yes	7	7 No	No	10
19						
19	1	Yes	7	2 Yes	Yes	14
19	2	No	7	7 Yes	No	14
19	3	No	5	5 Yes	No	0
19	4	Yes	4	4 Yes	Yes	14
19	5	Yes	4	4 Yes	Yes	2
19	6	No	4	4 Yes	No	2
19	7	Yes	7	7 Yes	Yes	14
19	8	Yes	6	6 Yes	Yes	13
19	9	Yes	7	7 Yes	Yes	14
19	10	Yes	7	7 Yes	Yes	14
19	11	Yes	7	7 Yes	Yes	4
20						
20	1	N/A or blar	7	7 Yes	No	0
20	2	Yes	7	7 Yes	No	0

20	3	Yes	5	5 Yes	No	0
20	4	Yes	5	5 Yes	No	0
20	6	Yes	4	4 Yes	No	9
20	7	Yes	7	7 Yes	Yes	14
20	8	No	7	7 Yes	No	0
20	9	Yes	7	7 Yes	No	0
20	10	Yes	7	7 Yes	Yes	14
20	11	Yes	4	4 Yes	Yes	7
21						
21	1	No	4	3 Yes	No	0
21	2	Yes	0	0 Yes	No	0
21	3	Yes	3	3 Yes	No	0
21	4	No	7	7 Yes	No	0
21	5	Yes	5	5 Yes	No	0
21	6	No	6	5 Yes	No	0
21	7	Yes	4	2 Yes	Yes	1
21	8	Yes	4	4 Yes	No	6
21	9	Yes	4	4 Yes	Yes	5
21	10	Yes	0	0 Yes	Yes	0
21	11	No	0	0 Yes	No	0
22						
22	1	N/A or blar	4	4 Yes	No	13
22	2	Yes	3	3 Yes	Yes	8
22	3	No	3	3 Yes	Yes	12
22	6	Yes	6	6 Yes	No	10
22	4	Yes	2	2 Yes	Yes	10
22	8	No	6	6 Yes	Yes	12
22	9	Yes	4	4 Yes	Yes	7
22	10	Yes	7	7 Yes	No	0
22	11	Yes	4	4 Yes	No	0
22	12	Yes	4	4 Yes	No	0
23						
23	1	No	7	7 Yes	No	0
23	2	Yes	4	4 Yes	Yes	10
23	3	No	7	7 Yes	Yes	14
23	5	N/A or blar	5	5 Yes	No	5
23	6	No	6	4 Yes	No	0
23	7	Yes	7	7 Yes	Yes	14
23	8	No	5	4 Yes	No	0
23	9	No	7	7 Yes	Yes	14
23	10	Yes	7	4 Yes	No	0
23	11	Yes	7	7 Yes	No	14
23	12	No	7	7 Yes	No	0
24						

24	1	No	3	4	Yes	No	0
24	3	Yes	4	4	Yes	No	0
24	4	No	2	0	Yes	No	0
24	5	No	5	5	Yes	Yes	4
24	6	Yes	6	6	Yes	Yes	10
24	7	No	6	6	Yes	Yes	12
24	8	No	7	7	Yes	No	14
24	9	No	7	7	Yes	No	14
24	10	Yes	7	7	Yes	No	14
24	11	Yes	5	7	Yes	Yes	0
24	12	Yes	5	5	Yes	Yes	14
25							
25	1	No	7	0	Yes	No	5
25	2	No	1	2	Yes	Yes	0
25	4	Yes	4	4	No	No	0
25	5	Yes	5	5	Yes	No	13
25	6	No	6	0	Yes	Yes	6
25	7	Yes	0	7	Yes	Yes	10
25	8	Yes	7	4	Yes	Yes	12
25	9	Yes	4	4	Yes	Yes	2
25	10	Yes	7	7	Yes	Yes	14
25	11	Yes	7	2	Yes	Yes	14
26							
26	1	Yes	7	7	No	No	0
26	2	Yes	7	7	Yes	No	0
26	3	N/A or blar	7	7	Yes	Yes	14
26	4	Yes	7	7	Yes	Yes	14
26	5	N/A or blar	7	7	Yes	Yes	10
26	6	No	5	4	Yes	No	10
26	7	No	6	6	Yes	Yes	11
26	8	Yes	7	7	Yes	No	10
26	9	Yes	7	7	Yes	Yes	14
26	10	Yes	7	4	Yes	No	0
26	11	Yes	7	4	Yes	No	0
26	12	No	7	7	Yes	No	0

rded
