# Supplementary material for: Prospective cohort study evaluating feasibility, acceptability, and clinical impact of diabetes self-management education in a PEN-Plus program in Southeastern Liberia
Source: PLOS Glob Public Health. 2025 Dec 15;5(12):e0005657. doi: 10.1371/journal.pgph.0005657 (PMC12704866; doi:10.1371/journal.pgph.0005657)
Supplement: Attachment — Submitted filename: replies author.docx [file pgph.0005657.s003.docx]

The topic of the manuscript is of interest in the LLMICs, despite the literature already available in the developed world. The concept of the study is relevant to clinical settings in LLMICs, but the execution has been less than satisfactory (probably related to the restrictions of COVID 19 pandemic) and the writing of the manuscript is voluminous for the limited work done. The number of participants is too small (3 providers and 26 persons with insulin dependent diabetes mellitus) for any meaningful conclusions to be drawn as well as for scalability and application to communities even in LLMICs. There are a few more issues that need to be addressed before this manuscript can be considered further.

I am intrigued as to how only 26 PLWIDD could represent all patients on insulin among a population of 172,202. The authors need to mention the prevalence of diabetes mellitus (both T1 & T2) in Liberia and the total number of patients on insulin in the studied area so that the readers put the study population in perspective.

The psychosocial assessment part has many inadequacies and rightly acknowledged by the authors. However, trying to present the results of that part and discussing its relevance would be misleading. Hence, it should be preferably deleted.

Specific points

Line 84 – 89

The description of PEN-Plus is confusing to the reader – PEN-Plus addresses severe NCDs like Type 1 DM, Rheumatic and congenital heart disease, sickle cell disease – the statement on lines 87 to 89 seems to suggest it is only for insulin dependent diabetes mellitus – needs to be better framed.

Line 159 to 161:

The authors state the following:

However, consistent with Essien *et* al in Nigeria[17], it was calculated that the study would have over 80% power using a two-tailed test with alpha value of 5% to detect a 1% decrease in HBA1c assuming a SD of 1.7 or less.

How would sample size be relevant when entire eligible population is recruited? How can sample size be calculated using one secondary outcome (HbA1c)?

Probably, this part can be deleted.

Line 167 to 178:

The DSME component needs further description – was the ‘provider’ training based on study specific modules or modules in public domain or some other source material?

Line 180

The authors state the following:

All PLWIDD were asked to come to clinic together on the same day each month.

What was the idea behind this – any specific advantage? How would this relate to up-scaling this project?

Primary Outcomes (Line 190 onwards) and Secondary Outcomes (Line 203 onwards)

The frequency/ timing of the measurement in relation to the study is mentioned for the secondary outcomes “HbA1c” and “Retention of provider and patient knowledge”. The same should be mentioned for other outcomes:

Acceptability

Adoption

Fidelity

Self-management behaviours (probably checked every clinic visit, but need to explicitly mention it)

Psychosocial wellbeing

Severe adverse events

Line 246 -247

The authors state the following:

Two standard clinical simulation scenarios were developed to assess growth in provider’s communication skills

What simulation scenarios are the authors referring to? Please describe both the scenarios.

Line 254 – 275

The mixed effects model needs clearer explanation including all symbols mentioned in the equation

Line 264 – 266

The authors state the following:

We developed a comprehensive provider assessment to assess DSME skills and knowledge of diabetes management designed to assess a preset list of competencies for diabetes management within PEN Plus.

This sentence is confusing to the reader with repetition of words like “assess” and “diabetes management”. Please simplify it and if necessary make two sentences.

With the description in the text, Table 2 is redundant and should be removed.

In each section of the results, the number of participants completing a particular visit for an outcome measure is different. Since the number of enrolled participants is small, further reduction of numbers makes the results very weak.

Line 417 – 419

For “provider knowledge”, the results are mentioned at baseline and 6 months. Please mention results at 12 months too.

There are a few grammatical/ typo errors that need to be corrected.

It is preferable to use the word “diabetes mellitus” rather than “diabetes” at least at the beginning of the manuscript.

Grammatical/typo errors/ inconsistency in language:

Few examples:

Line 48

The authors make the following statement:

People living with insulin dependent diabetes (PLWIDD) in low and lower-middle income

countries, specifically….

The abbreviation “LLMICs” should be provided here

On a similar note, the abbreviation, SSA is not expanded in the manuscript – sub-Saharan Africa

Line 139

The authors make the following statement:

Phase 2 started August 2021.

The statement **“**Phase 2 started August 2021” is abrupt, informal and grammatically inappropriate for a manuscript and should be better framed

Similarly, Line 162

The authors make the following statement:

Study recruitment started July 12, 2021 and completed August 20, 2021

The statement “Study recruitment started July 12, 2021 and completed August 20, 2021” –needs to be properly framed.

The word Hemoglobin A1c has been written differently in the manuscript - “HbA1c” at some places and “HBA1c” at others – please maintain uniformity.

Line 374 – 375

The authors make the following statement:

However, the frequency providers reviewed SMART goals from the previous visit varied from 39.1% to 100%

The statement should be…… However, the frequency ***at which*** providers reviewed SMART goals from the previous visit varied from 39.1% to 100%

The words in italics should be added.

Please ensure other such errors are corrected.
