# Supplementary material for: Prospective cohort study evaluating feasibility, acceptability, and clinical impact of diabetes self-management education in a PEN-Plus program in Southeastern Liberia
Source: PLOS Glob Public Health. 2025 Dec 15;5(12):e0005657. doi: 10.1371/journal.pgph.0005657 (PMC12704866; doi:10.1371/journal.pgph.0005657)
Supplement: Attachment — Submitted filename: Response to Reviewers.docx [file pgph.0005657.s005.docx]

To whom it may concern:

Please see responses to reviewers below, in blue text.

Journal Requirements:

1. Please include a complete copy of PLOS’ questionnaire on inclusivity in global research in your revised manuscript. Our policy for research in this area aims to improve transparency in the reporting of research performed outside of researchers’ own country or community. The policy applies to researchers who have travelled to a different country to conduct research, research with Indigenous populations or their lands, and research on cultural artefacts. The questionnaire can also be requested at the journal’s discretion for any other submissions, even if these conditions are not met. Please find more information on the policy and a link to download a blank copy of the questionnaire here: https://journals.plos.org/globalpublichealth/s/best-practices-in-research-reporting. Please upload a completed version of your questionnaire as Supporting Information when you resubmit your manuscript.

Questionnaire will be uploaded with this resubmission.

2. In the online submission form, you indicated that [Data are available upon reasonable request. Deidentified data are available upon reasonable request from the corresponding author (GF) at gferrari@bwh.harvard.edu.].

All PLOS journals now require all data underlying the findings described in their manuscript to be freely available to other researchers, either

a. In a public repository,

b. Within the manuscript itself, or

c. Uploaded as supplementary information.

This policy applies to all data except where public deposition would breach compliance with the protocol approved by your research ethics board. If your data cannot be made publicly available for ethical or legal reasons (e.g., public availability would compromise patient privacy), please explain your reasons by return email and your exemption request will be escalated to the editor for approval. Your exemption request will be handled independently and will not hold up the peer review process, but will need to be resolved should your manuscript be accepted for publication. One of the Editorial team will then be in touch if there are any issues.

Data will be uploaded as supplementary information with this resubmission.

Reviewer 1

The topic of the manuscript is of interest in the LLMICs, despite the literature already available in the developed world. The concept of the study is relevant to clinical settings in LLMICs, but the execution has been less than satisfactory (probably related to the restrictions of COVID 19 pandemic) and the writing of the manuscript is voluminous for the limited work done. The number of participants is too small (3 providers and 26 persons with insulin dependent diabetes mellitus) for any meaningful conclusions to be drawn as well as for scalability and application to communities even in LLMICs. There are a few more issues that need to be addressed before this manuscript can be considered further.

I am intrigued as to how only 26 PLWIDD could represent all patients on insulin among a population of 172,202. The authors need to mention the prevalence of diabetes mellitus (both T1 & T2) in Liberia and the total number of patients on insulin in the studied area so that the readers put the study population in perspective.

Thank you for your comment. Maryland county is an extremely rural, remote and impoverished region of Liberia, that is sadly underserved by medical clinics. The PEN-Plus clinic was established in 2017, and at the point, there was almost no care available anywhere for T1D. As a result, anyone who had a serious condition would die without being able to access care. Once the PEN-Plus clinic was established, one of the main activities was case-finding. Unfortunately, due to the remoteness, rural nature, and extreme poverty, some people are still missed. These are the two main centers where diabetes is treated in Maryland county, and this is the entire population of people living with type 1 diabetes in these centers. We acknowledge the low prevalence respective to the population of the county-but unfortunately there is a high mortality rate among undiagnosed people. The program continues to do its best for case-finding, but unfortunately staffing and funding is a continual problem.

The psychosocial assessment part has many inadequacies and rightly acknowledged by the authors. However, trying to present the results of that part and discussing its relevance would be misleading. Hence, it should be preferably deleted.

We agree about the limitations of the psychosocial wellbeing data. We have removed it from the manuscript and updated the description of protocol deviations:

“Study protocol dictated data collection on psychosocial wellbeing using the Patient Health Questionnaire-9 (PHQ-9) and Problem Areas In Diabetes (PAID-5). While some instances were collected, data quality was poor in part due to missing data and in part due to inadvertent use of the PHQ-2, which has not been validated in Liberia. Therefore, these data are not included in this manuscript.”

And have updated the discussion:

“Though we were not able to report on psychosocial outcomes from this study due to data quality issues, we encourage inclusion of these outcomes in future research.”

Specific points

Line 84 – 89

The description of PEN-Plus is confusing to the reader – PEN-Plus addresses severe NCDs like Type 1 DM, Rheumatic and congenital heart disease, sickle cell disease – the statement on lines 87 to 89 seems to suggest it is only for insulin dependent diabetes mellitus – needs to be better framed.

Description of PEN-Plus has been updated:

“To improve care for PLWIDD living in rural areas, in 2017 Liberia initiated the PEN-Plus strategy of care for severe chronic conditions beginning with Maryland County in the impoverished Southeastern part of the country. PEN-Plus (the Package of Essential Noncommunicable Disease Interventions – Plus) is a comprehensive strategy to decentralize care for multiple severe NCDs including insulin-dependent diabetes by training and equipping mid-level providers to manage several chronic conditions using shared infrastructure”

Line 159 to 161:

The authors state the following:

However, consistent with Essien *et* al in Nigeria[17], it was calculated that the study would have over 80% power using a two-tailed test with alpha value of 5% to detect a 1% decrease in HBA1c assuming a SD of 1.7 or less.

How would sample size be relevant when entire eligible population is recruited? How can sample size be calculated using one secondary outcome (HbA1c)?

Probably, this part can be deleted.

This section has been deleted.

Line 167 to 178:

The DSME component needs further description – was the ‘provider’ training based on study specific modules or modules in public domain or some other source material?

Additional clarification about the DSME training has been added:

Training curriculum was adapted from a course for Nurse Practitioner students entitled “Behavioral Approaches to Diabetes Across the Lifespan” at the University of California, San Francisco{McGrath, 2023}. New training content was developed for this study due to the need to adapt to local context.

Line 180

The authors state the following:

All PLWIDD were asked to come to clinic together on the same day each month.

What was the idea behind this – any specific advantage? How would this relate to up-scaling this project?

Participants were asked to come to clinic on the same day each month because this allowed for a team-based approach to care with mental health present, as well as exchange of experience and mutual support between patients.

Text has been updated:

“All PLWIDD were asked to come to clinic together on the same day each month because this allowed for a team-based approach to care with mental health present, as well as exchange of experience and mutual support between patients.”

Primary Outcomes (Line 190 onwards) and Secondary Outcomes (Line 203 onwards)

The frequency/ timing of the measurement in relation to the study is mentioned for the secondary outcomes “HbA1c” and “Retention of provider and patient knowledge”. The same should be mentioned for other outcomes:

Acceptability

Adoption

Fidelity

Self-management behaviours (probably checked every clinic visit, but need to explicitly mention it)

Psychosocial wellbeing – has been removed from paper per reviewer recommendations

Severe adverse events

Timing of data collection has been added for missing outcomes.

Line 246 -247

The authors state the following:

Two standard clinical simulation scenarios were developed to assess growth in provider’s communication skills

What simulation scenarios are the authors referring to? Please describe both the scenarios.

Description of the scenarios has been added:

“One was a standardized patient for a typical diabetes visit, while the other was a patient with alcohol use disorder and diabetes.”

Line 254 – 275

The mixed effects model needs clearer explanation including all symbols mentioned in the equation

Text has been edited to clarify:

To account for repeated measures, estimated changes in HbA1c calculated from baseline using a mixed effects model are shown below. HbA1c was estimated for individual i at time t with fixed effects (β_1-3_) on time indicators (T3, T6, and T12) for the three-, six-, and 12-month measurements, a fixed intercept for baseline (β_0_), and a random intercept for individuals (δ_i_).

$${HbA1c}_{i,t}=\beta_{0}+\beta_{1}{T3}_{i,t}+\beta_{2}{T6}_{i,t}+\beta_{3}{T12}_{i,t}+\delta_{i}+\varepsilon_{it}$$

Line 264 – 266

The authors state the following:

We developed a comprehensive provider assessment to assess DSME skills and knowledge of diabetes management designed to assess a preset list of competencies for diabetes management within PEN Plus.

This sentence is confusing to the reader with repetition of words like “assess” and “diabetes management”. Please simplify it and if necessary make two sentences.

Section has been edited as follows, including additional clarifications recommended by a different reviewer:

“A 41-question multiple choice exam was developed to assess DSME skills and knowledge of diabetes management. This assessment was designed to match pre-existing competencies for diabetes management within PEN-Plus, so to date has not been externally validated. Domains included psychosocial management, age-appropriate education, basic diabetes pathophysiology and management, glucose levels, glucose monitoring, insulin administration, insulin dosing, nutrition and diet, physical activity, and complications screening.”

With the description in the text, Table 2 is redundant and should be removed.

Thank you for your comment. Respectfully, we would like to keep the table in the text so that readers have easy access to the data in a clearer format.

In each section of the results, the number of participants completing a particular visit for an outcome measure is different. Since the number of enrolled participants is small, further reduction of numbers makes the results very weak.

We understand that this is a limitation of the study. Given it was a pilot study and the entire population of PLWIDD were enrolled, we were not able to control missing data that occurred if participants missed visits, which is common in this setting due to the many social stressors patients are facing.

Line 417 – 419

For “provider knowledge”, the results are mentioned at baseline and 6 months. Please mention results at 12 months too.

We were unable to complete the assessment month 12 due to travel restrictions. The manuscript has been updated to clarify:

“Due to travel restrictions, provider knowledge was not assessed during month 12.”

There are a few grammatical/ typo errors that need to be corrected.

It is preferable to use the word “diabetes mellitus” rather than “diabetes” at least at the beginning of the manuscript.

Text has been updated.

Grammatical/typo errors/ inconsistency in language:

Few examples:

Line 48

The authors make the following statement:

People living with insulin dependent diabetes (PLWIDD) in low and lower-middle income

countries, specifically….

The abbreviation “LLMICs” should be provided here

Abbreviation has been added.

On a similar note, the abbreviation, SSA is not expanded in the manuscript – sub-Saharan Africa

This has been corrected.

Line 139

The authors make the following statement:

Phase 2 started August 2021.

The statement **“**Phase 2 started August 2021” is abrupt, informal and grammatically inappropriate for a manuscript and should be better framed

Text has been updated in this paragraph for clarity:

“During this phase, clinicians (cohort one) who were currently providing care for PLWIDD were enrolled and received training on how to administer DSME. Phase 2 began in August 2021. In phase two, PLWIDD (cohort two) were enrolled and received structured DSME for one year administered by providers.”

Similarly, Line 162

The authors make the following statement:

Study recruitment started July 12, 2021 and completed August 20, 2021

The statement “Study recruitment started July 12, 2021 and completed August 20, 2021” –needs to be properly framed.

Text has been updated:

“Study recruitment occurred between July 12, 2021 and August 20, 2021.”

The word Hemoglobin A1c has been written differently in the manuscript - “HbA1c” at some places and “HBA1c” at others – please maintain uniformity.

Manuscript has been updated for uniformity.

Line 374 – 375

The authors make the following statement:

However, the frequency providers reviewed SMART goals from the previous visit varied from 39.1% to 100%

The statement should be…… However, the frequency ***at which*** providers reviewed SMART goals from the previous visit varied from 39.1% to 100%

The words in italics should be added.

Manuscript has been updated with suggested edit.

Please ensure other such errors are corrected.

**Reviewer 2**

General Comments:

This manuscript presents a relevant and well-structured study assessing the feasibility and clinical impact of a diabetes self-management education (DSME) intervention in a resource-constrained setting. The focus on PEN-Plus implementation and involvement of a patient advisory board is commendable. However, a few clarifications and elaborations are needed to enhance the rigor and interpretability of the findings.

Specific Comments:

1. Knowledge assessment tool:

A brief description of the diabetes knowledge assessment tool should be included—specifying the number of items, the domains covered, scoring method, maximum possible score and whether the tool was validated or adapted for this context.

Section has been edited as follows, including additional clarifications recommended by a different reviewer:

“A 41-question multiple choice exam was developed to assess DSME skills and knowledge of diabetes management. This assessment was designed to match pre-existing competencies for diabetes management within PEN-Plus, so to date has not been externally validated. Domains included psychosocial management, age-appropriate education, basic diabetes pathophysiology and management, glucose levels, glucose monitoring, insulin administration, insulin dosing, nutrition and diet, physical activity, and complications screening.”

2. Data analysis section:

The Data Analysis section currently mentions only the software used. Please elaborate on the analytical approach adopted for key outcomes, including model specifications, handling of missing data, and any sensitivity analyses conducted.

The software used is reported at the beginning of the data analysis section because it was the same throughout the analysis. Because each outcome had a different analysis method, they are listed separately under the subheadings, shared below:

“HbA1c

Due to procurement issues with HbA1c cartridges, no HbA1cs were checked at month nine. Some individuals were missing measurements for particular time points; estimates for all available data and for the subset of individuals without missing measurements are shown at any time point. To account for repeated measures, estimated changes in HbA1c calculated from baseline using a mixed effects model are shown below. HbA1c was estimated for individual i at time t with fixed effects (β_1-3_) on time indicators (T3, T6, and T12) for the three-, six-, and 12-month measurements, a fixed intercept for baseline (β_0_), and a random intercept for individuals (δ_i_).

$${HbA1c}_{i,t}=\beta_{0}+\beta_{1}{T3}_{i,t}+\beta_{2}{T6}_{i,t}+\beta_{3}{T12}_{i,t}+\delta_{i}+\varepsilon_{it}$$

Knowledge Assessments

A 41-question multiple choice exam was developed to assess DSME skills and knowledge of diabetes management. This assessment was designed to match pre-existing competencies for diabetes management within PEN-Plus, so to date has not been externally validated. Domains included psychosocial management, age-appropriate education, basic diabetes pathophysiology and management, glucose levels, glucose monitoring, insulin administration, insulin dosing, nutrition and diet, physical activity, and complications screening.

Patient knowledge assessments were developed in alignment with the ADCES seven self-care behaviors(9) and adapted with guidance from local providers to local context in Liberia. Because most patients have limited literacy, providers read the questions to the patients and marked their answers.

The mean and standard deviation of the scores on assessments were calculated at each time point. To account for repeated measures, score changes were estimated from baseline using a mixed effects model shown below, where Y is the knowledge score for individual i at time t, T6 and T12 are indicators for the six- month and 12-month assessments, respectively, and δ_i_ is a random intercept for individuals. Coefficients β_1-2_ are fixed coefficients, and β_0_ is a fixed intercept.

$Y_{i,t}=\beta_{0}+\beta_{1}{T6}_{i,t}+\beta_{2}{T12}_{i,t}+\delta_{i}+\varepsilon_{i,t}$”

3. Statistical modelling:

In the description of the repeated measures mixed-effect model, please specify which variables were treated as fixed effects and which were modelled as random effects.

Text has been edited to clarify:

To account for repeated measures, estimated changes in HbA1c calculated from baseline using a mixed effects model are shown below. HbA1c was estimated for individual i at time t with fixed effects (β_1-3_) on time indicators (T3, T6, and T12) for the three-, six-, and 12-month measurements, a fixed intercept for baseline (β_0_), and a random intercept for individuals (δ_i_).

$${HbA1c}_{i,t}=\beta_{0}+\beta_{1}{T3}_{i,t}+\beta_{2}{T6}_{i,t}+\beta_{3}{T12}_{i,t}+\delta_{i}+\varepsilon_{it}$$

4. Incentives for participation:

It would be helpful to clarify whether any monetary or non-monetary incentives were provided to patient advisory board members and to the providers for participating in home visits.

The following sentence has been added for clarity:

PAB members received transportation assistance to attend meetings but were not compensated additionally for their time.

5. Frequency of self-management behaviors:

There appears to be a lack of internal coherence in the reporting of self-management behaviors. For example:

“Participants brought glucometers to clinic at 87.1% of encounters. The frequency stayed consistent from month one (69.2%, n=26) to month 12 (64.7%, n=12).”

“Participants brought logbooks to clinic at 59.6% of encounters. This also stayed consistent from month one (42.3%, n=26) to month 12 (47.1%, n=12).”

These statements suggest higher frequency and then month wise frequency is reported as lower. Please revise for clarity and internal consistency.

Text has been revised to include only the aggregate number of times that glucometers and logbooks were brought to clinic:

“Participants brought glucometers to clinic at 87.1% of encounters and brought logbooks to clinic 59.6% of encounters.”

6. Referrals after PAID and PHQ screening:

The manuscript notes 26 referrals after psychosocial screening. Please clarify whether this figure refers to 26 unique patients or multiple referrals for some individuals. This information should also be included in the Results section.

Per recommendations of another reviewer, the psychosocial data (PAID and PHQ screening) has been removed from the manuscript due to poor data quality.

**Reviewer 3**

This is a well-conceived, contextually relevant, and competently executed pilot study assessing the feasibility, acceptability, and preliminary impact of Diabetes Self-Management Education (DSME) among people living with insulin-dependent diabetes (PLWIDD) in rural Liberia. The manuscript addresses a critical gap in implementation science, particularly in the context of Type 1 diabetes care in low-resource settings, making it both timely and valuable. However, there are several areas where clarity and depth can be improved. Detailed comments are provided below:

• The Introduction is generally well-written. However, the description of the PEN-Plus model could be expanded for readers who may not be familiar with it.

The description of PEN-Plus has been updated, inclusive of comments from other reviewers:

“To improve care for PLWIDD living in rural areas, in 2017 Liberia initiated the PEN-Plus strategy of care for severe chronic conditions beginning with Maryland County in the impoverished Southeastern part of the country. PEN-Plus (the Package of Essential Noncommunicable Disease Interventions – Plus) is a comprehensive strategy to decentralize care for multiple severe NCDs including insulin-dependent diabetes by training and equipping mid-level providers to manage several chronic conditions using shared infrastructure”

• The study is described as a “prospective dual-cohort”, but only one cohort (PLWIDD receiving DSME) is presented. Please clarify what is meant by “dual-cohort” or revise to simply state “prospective cohort study.”

We describe the study as dual-cohort considering providers as cohort one, and patients as cohort two. The text has been updated to clarify:

“Phase one began July 2021 and continued for 12 months. During this phase, clinicians (cohort one) who were currently providing care for PLWIDD were enrolled and received training on how to administer DSME. Phase 2 began in August 2021. In phase two, PLWIDD (cohort two) were enrolled and received structured DSME for one year administered by providers.”

• The reference to behaviour change models (line 104) is vague. Please specify which theoretical model(s) guided the DSME intervention design.

Theoretical models have been specified in the text:

“The theoretical basis for DSME draws from several behavior change models, including the health belief model(12), social cognitive theory(13), and the transtheoretical model(14).”

• Clarify whether the knowledge assessment tools used were previously validated instruments or developed specifically for this study.

Section has been edited as follows, including additional clarifications recommended by a different reviewer:

“A 41-question multiple choice exam was developed to assess DSME skills and knowledge of diabetes management. This assessment was designed to match pre-existing competencies for diabetes management within PEN-Plus, so to date has not been externally validated. Domains included psychosocial management, age-appropriate education, basic diabetes pathophysiology and management, glucose levels, glucose monitoring, insulin administration, insulin dosing, nutrition and diet, physical activity, and complications screening.”

• Provide more detail on the Patient Advisory Board (PAB) mentioned in line 134. What was the composition of the board, and what roles did they play? Were there specific criteria for selecting patient participants?

Additional information about the PAB has been provided in the results section:

“The PAB consisted of 12 members who were either PLWIDD or family members. Participants were selected by providers based on active involvement in the clinic. PAB members received transportation assistance to attend meetings but were not compensated additionally for their time.”

• The lack of a comparison group limits the ability to draw causal inferences. While this limitation is acknowledged, the use of a matched pre-post design or a stepped-wedge approach could have strengthened the findings. Consider explicitly discussing this in the Discussion section.

A sentence has been added in the discussion:

“Repetition of this study with a more robust design and larger sample size would be beneficial.”

• The observed reduction in HbA1c at 3 months (from 12.0% to 9.1%) followed by an increase at 6 and 12 months (10.9%) warrants further discussion. What might explain this trajectory, and what are the implications for intervention sustainability?

Some postulations about reasons for this trajectory have been added to the discussion:

“Of note, the largest observed decrease was in month three, followed by an increase in month six. This may have been the result of seasonal variability in food security, which can negatively impact diabetes management or from fatigue in adherence to protocols from providers or patients over time. Due to the small sample size, more robust studies will be needed to confirm these results.”

• The modelled mean HbA1c reduction of 1.1% (95% CI: 0.0 to 2.0) approaches significance but the wide confidence interval suggests uncertainty. It is important to caution readers that while the trend is encouraging, more robust data from larger samples are needed.

A sentence has been added to the discussion:

“Due to the small sample size, more robust studies will be needed to confirm these results.”

• The integration of qualitative quotes is effective in illustrating participant experiences. However, consider reducing the number of direct quotes in the main text to improve readability and focus.

Two of the direct quotes have been removed from the main text.

• Where possible, report effect sizes with confidence intervals consistently (e.g., for knowledge scores, frequency of glucose monitoring) to aid interpretation.

Effect size and confidence intervals are reported for patient knowledge scores, and text has been revised for clarity. Effect sizes for provider knowledge scores are not reported as the number was very small (n=3), though this has been added to the text. Confidence intervals have been added to frequency of blood glucose monitoring and missed insulin injections:

“Average blood glucose checks increased from 4.0 (95% CI 1.93, 6.23) checks per week month one, to 7.2 (95% CI 3.56, 10.79) checks per week month 12 (Fig 2). Average missed insulin injections decreased from 3.9 (95% CI 2.51, 6.03) in month one, to 0.82 (95% CI -0.14, 1.79) in month 12 (Fig 2). “
